# Modulation of Microglial Activity by Rho-Kinase (ROCK) Inhibition as Therapeutic Strategy in Parkinson’s Disease and Amyotrophic Lateral Sclerosis

**DOI:** 10.3389/fnagi.2017.00094

**Published:** 2017-04-04

**Authors:** Anna-Elisa Roser, Lars Tönges, Paul Lingor

**Affiliations:** ^1^Department of Neurology, University Medicine GöttingenGöttingen, Germany; ^2^DFG Cluster of Excellence Nanoscale Microscopy and Molecular Physiology of the Brain (CNMPB), University Medicine GöttingenGöttingen, Germany; ^3^Department of Neurology, Ruhr-Universität BochumBochum, Germany

**Keywords:** microglial polarization, Parkinson’s disease, amyotrophic lateral sclerosis, ROCK, ROCK inhibition, neuroinflammation

## Abstract

Neurodegenerative diseases are characterized by the progressive degeneration of neurons in the central and peripheral nervous system (CNS, PNS), resulting in a reduced innervation of target structures and a loss of function. A shared characteristic of many neurodegenerative diseases is the infiltration of microglial cells into affected brain regions. During early disease stages microglial cells often display a rather neuroprotective phenotype, but switch to a more pro-inflammatory neurotoxic phenotype in later stages of the disease, contributing to the neurodegeneration. Activation of the Rho kinase (ROCK) pathway appears to be instrumental for the modulation of the microglial phenotype: increased ROCK activity in microglia mediates mechanisms of the inflammatory response and is associated with improved motility, increased production of reactive oxygen species (ROS) and release of inflammatory cytokines. Recently, several studies suggested inhibition of ROCK signaling as a promising treatment option for neurodegenerative diseases. In this review article, we discuss the contribution of microglial activity and phenotype switch to the pathophysiology of Parkinson’s disease (PD) and Amyotrophic lateral sclerosis (ALS), two devastating neurodegenerative diseases without disease-modifying treatment options. Furthermore, we describe how ROCK inhibition can influence the microglial phenotype in disease models and explore ROCK inhibition as a future treatment option for PD and ALS.

## Introduction

The idea that neurodegenerative disorders (NDD) are primarily caused by a particular susceptibility of neuronal subpopulations to damaging insults is challenged by the observation that a pronounced infiltration of microglia into affected brain regions is a common hallmark of NDD. The discovery of a microglial phenotype switch from an immune-suppressive neuroprotective to a pro-inflammatory neurotoxic phenotype emphasizes the importance of a microglial contribution to the formation and course of NDD. One major regulator of microglial activity is the Rho-kinase (ROCK) signaling pathway. ROCK is a serine/threonine kinase that is expressed as two homologs, ROCK1 and ROCK2. The two isoforms share similar structure and function, but show differences in their abundance. ROCK1 is the dominant form in liver, lung, testes, blood and the immune system, whereas ROCK2 is dominant in the brain and muscles (Nakagawa et al., 1996; Hashimoto et al., 1999). ROCK activity is regulated by its upstream regulators, the Rho-GTPases RhoA and RhoC, which belong to the Ras-superfamily (Leung et al., 1996; Hashimoto et al., 1999). Active ROCK phosphorylates numerous downstream targets which are involved in regulation of cell shape and motility as well as apoptosis and cell survival. In microglial cells the ROCK pathway is involved in regulation of migration, phagocytosis and release of inflammatory cytokines and thus mediates the microglial phenotype (Yan et al., 2012; Borrajo et al., 2014a). Additionally, there is accumulating evidence that ROCK is increased in microglial cells in Parkinson’s disease (PD) and Amyotrophic lateral sclerosis (ALS; Conti et al., 2014; Saal et al., 2017). Thus, inhibition of the ROCK pathway could be a promising treatment option for NDD. In this review article, we show how ROCK inhibition can influence the microglial phenotype, discuss the microglial contribution to the pathophysiology of PD and ALS and explore ROCK inhibition as a treatment option for PD and ALS.

## Microglial Polarization and Phenotypes

Microglia are the resident innate immune cells with a macrophage-like capacity in the otherwise immune-privileged central nervous system (CNS; Kettenmann et al., 2011). Microglial cells fulfill a broad variety of functions and can commit to different reactive phenotypes (Schwartz et al., 2006; Hanisch and Kettenmann, 2007). In the healthy brain microglia occur in a so-called “resting” state with a ramified phenotype, which presents highly motile processes that continuously scan their environment. The processes can detect even slight aberrations in neural signal propagation and in occurrence and quantity of extracellular molecules. Thus, “resting” microglia serve mainly in the surveillance and maintenance of the CNS homeostasis (Kettenmann et al., 2011; Wolf et al., 2017). Under pathological conditions microglia can be “activated”, e.g., by infectious agents, tissue damage or functional modifications of neighboring neurons. This is accompanied by a drastic morphological change towards an ameboid phenotype and an altered expression of surface molecules and releasable factors (Kettenmann et al., 2011; Hu et al., 2015) that is strongly dependent on the brain region and the nature of the activating agent. It was shown that microglia isolated from different brain regions and the spinal cord differ in the severity of a pro-inflammatory response (Baskar Jesudasan et al., 2014). Activated microglia can be classified according to the peripheral macrophage classification system in “M1” and “M2” microglia, even though this oversimplified model only represents two extreme activation states and it has become clear that microglial activation is a highly dynamic process with more than the two polarities (Franco and Fernández-Suárez, 2015; Tang and Le, 2016; Wolf et al., 2017). The “M1” phenotype is associated with the release of proinflammatory cytokines (e.g., TNF-α, IL-1β), nitric oxide (NO) and reactive oxygen species (ROS) and bears the risk of harming neuronal cells with prolonged activation (Hanisch and Kettenmann, 2007; Tang and Le, 2016). The “M2” phenotype provides a rather neuroprotective environment by the release of anti-inflammatory cytokines (e.g., IL-4, IL-10, IL-13) or growth factors such as TGF-β and promotes tissue repair and regeneration. A growing body of evidence suggests different “M2” subpopulations with distinct biological functions (Hu et al., 2015; Tang and Le, 2016).

## The Role of Rock Activity for the Inflammatory Response of Microglia

ROCK activity plays a crucial role in microglial activation. It has been shown that it is involved in the regulation of key features of microglial activitiy like migration, phagocytosis and release of cytokines and chemokines (Barcia et al., 2012; Yan et al., 2012; Borrajo et al., 2014a). Microglia are migratory cells that can travel along chemokine gradients towards a site of infection or injury. Motility is a process that mainly reflects the dynamic organization of the actin cytoskeleton and that is critically controlled by the Rho family of small GTPases (Welch and Mullins, 2002). ROCK maintains stress fibers and focal adhesions, whereas Rac organizes the formation of filopodia, both necessary structures for migration (Nobes and Hall, 1995). For phagocytotic cells like microglia, the remodeling of the actin cytoskeleton is not only important for migration, but also enables them to engulf particles and cellular debris. Even though phagocytosis is initiated by different receptors, downstream they all lead to activation of Rho GTPases for remodeling of the actin cytoskeleton (Chimini and Chavrier, 2000). As migration and phagocytosis are strongly dependent on the actin cytoskeleton the involvement of ROCK activity in these processes is not surprising. Interestingly, however, ROCK signaling plays also a role in microglial release of cytokines and chemokines, a process that is initiated by binding of ligands to specific surface receptors, e.g., purinergic receptors, toll-like receptors (TLRs) or angiotensin type receptors (Olson and Miller, 2004; Inoue, 2006; Rodriguez-Perez et al., 2015). Activation of purinergic receptors for extracellular nucleotides leads to microglial motility, chemotaxis and release of cytokines, NO and superoxides. Downstream of purinergic receptors different effector molecules are activated, among them JNK and MAPK signaling pathways and ROCK (Erb et al., 2006; Färber and Kettenmann, 2006). Another important family of receptors for the activation of microglia are TLRs. They have a variety of downstream targets including the RhoA-ROCK signaling pathway (Oda and Kitano, 2006). Microglial release of cytokines and chemokines is also mediated by angiotensin type receptors by activation of the nicotinamide adenine dinucleotide phosphate (NADPH)-oxidase complex and the ROCK signaling pathway (Rodriguez-Perez et al., 2015). There are several studies employing inhibition of ROCK that emphasize the significant role of this pathway for the microglial activation process. In a rat model of neuropathic pain, ROCK inhibition prevented morphological changes in microglia as well as microglia-neuron interactions (Tatsumi et al., 2015). In a mouse model for hypoxia and reoxygenation it was shown that ROCK inhibition by Fasudil led to a reduced expression of microglial inducible nitric oxide synthase (iNOS). Furthermore, treatment with the isoquinoline-type ROCK-inhibitor Fasudil reduced the microglial release of the pro-inflammatory factors NO, IL-1β, IL-6 and TNF-α (Ding et al., 2010). Similar findings were made in the rat retina (Tura et al., 2009). Additionally, there is strong evidence that the ROCK pathway is involved in modulation of the microglial phenotype. Even though there are no studies showing that ROCK activity is increased in a certain microglial phenotype, there are reports from different models showing that inhibition of ROCK leads to a phenotypic shift from “M1” towards “M2” microglia, suggesting that ROCK activation is necessary for maintenance of the pro-inflammatory “M1” phenotype. These phenotypic shifts were associated with a decrease in the release of the pro-inflammatory factors NO, IL-1β, IL-6 and TNF-α and increased the release of the anti-inflammatory factor IL-10 in cultured microglia (Ding et al., 2010). A similar suppression of the pro-inflammatory cytokines was observed in a mouse model after Fasudil-treatment (Tönges et al., 2014). Taken together, there is robust evidence that ROCK activity plays an important role in activation of microglia and the determination of its reactive phenotype.

## Microglial Activity in PD

PD is the second most common neurodegenerative disorder worldwide with a prevalence of about 0.3% in industrialized countries (Dexter and Jenner, 2013). One of its characteristics is a progressive degeneration of the dopaminergic nigrostriatal projections and their cell bodies in the substantia nigra. This leads to a lack of striatal dopamine and imbalanced basal ganglia signaling, causing severe motor deficits (Jankovic, 2008). Approximately 5% of all PD cases are inherited and caused by mutations in different genes or duplications/triplications of the α-synuclein locus. However, the majority of the PD cases occurs sporadically without identifiable cause (Dexter and Jenner, 2013).

There are numerous *in vitro* and animal studies suggesting a link between the loss of dopaminergic neurons and activation of microglia in the substantia nigra in PD. In animals treated with the neurotoxins 6-hydroxydopamine (6-OHDA) or 1-Methyl-4-phenyl-1,2,3,6-tetrahydropyridin (MPTP) an increased microglial infiltration and activation has been described (Akiyama and McGeer, 1989; Członkowska et al., 1996; Cicchetti et al., 2002; Gao et al., 2002; Noelker et al., 2013). Similar observations could be made in monkeys treated with MPTP. Here, a prolonged activation of microglia was observed even 1 year after MPTP treatment (Barcia et al., 2004). Interestingly, the postmortem tissues of human MPTP users showed also a prolonged activation of reactive microglia, even years after the acute intoxication (Langston et al., 1999). Furthermore, aggregated α-synuclein, one of the main hallmarks of PD, released from dying neurons leads to a microglial activation towards the pro-inflammatory “M1” phenotype (Zhang et al., 2005; Reynolds et al., 2008; Lee et al., 2010). It is possible to visualize reactive microglia *in vivo* by positron emission topography (PET) employing radiotracers that bind to surface structures of activated microglia, such as the isoquinoline-derivative and translocator protein (TSPO)-ligand [^11^C]-PK11195 (Bartels and Leenders, 2007). Different PET studies could confirm an increased microglial activation in the midbrain of PD patients that was correlated to disease progression (Ouchi et al., 2005; Gerhard et al., 2006; Koshimori et al., 2015). This goes in line with a study reporting that the pro-inflammatory cytokines TNFα, IL-6 and IL-1β were elevated in the cerebrospinal fluid (CSF) of PD patients (Qin et al., 2016). So far, all evidence on hand points towards an increased microglial activation with a pro-inflammatory “M1” phenotype that might contribute to PD progression. Little is known about the “M2” phenotype in PD.

**Figure 1:**
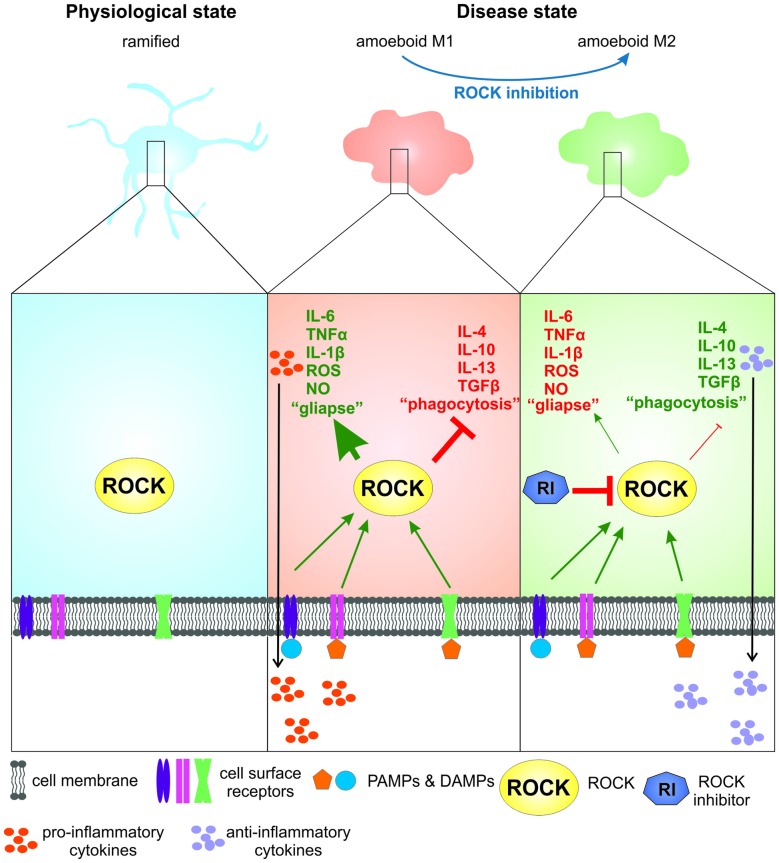
**The role of Rho-kinase (ROCK) activity and ROCK inhibition in microglia in Parkinson’s disease (PD) and amyotrophic lateral sclerosis (ALS).** In their physiological “ramified” stage microglia are continuously scanning their environment. For simplification, constitutive ROCK activity is disregarded in this illustration. Upon binding of pathogen- or danger associated molecular patterns (PAMPs or DAMPs; e.g., α-synuclein) to cell surface receptors microglia are activated, showing a “M1” phenotype that is associated with increased ROCK activity. This leads to a drastic change in morphology towards an ameboid shape, release of pro-inflammatory cytokines and chemokines and increased formation of microglia-neuron contacts (“gliapses”). Upon ROCK inhibition by a ROCK inhibitor the microglial phenotype changes towards a more “M2”-like phenotype, leading to the release of anti-inflammatory cytokines and growth factors and less “gliapse” formation.

Abnormal activity of ROCK associated with an increased inflammatory response was demonstrated in the substantia nigra of the MPTP mouse model for PD (Villar-Cheda et al., 2012). Additionally, our group could show that in postmortem tissue of PD patients compared to age-matched controls ROCK expression is increased in glial cells (Saal et al., 2017). It has been shown that, as the two phenotypes can transit into each other, treatment with a ROCK inhibitor skews “M1” toward “M2” microglia in experimental PD models and thus, is a promising therapeutic option for the treatment of PD (Zhao et al., 2015; He et al., 2016; summarized in Figure 1).

## Microglial Activation in ALS

ALS is a chronic progressive NDD with a fatal disease course. Because of the involvement of upper and lower motoneurons with subsequent impairment of the CNS corticopyramidal tract and peripheral motor axons patients suffer from progressive muscle weakness and paralysis, which ultimately leads to death within 3–5 years. The large majority of ALS is sporadic but there are also familial cases which have a genetic predisposition (e.g., mutations in the SOD1 gene or DNA repeat expansions in C9orf72; Paez-Colasante et al., 2015).

The immune system and neuroinflammation are strongly implicated in ALS progression and comprise microglial activation, T-cell-independent macrophage activation, monocyte recruitment to diseased tissue and dysregulation of immune-related genes in human ALS patients (Henkel et al., 2013; Philips and Rothstein, 2014). This neuroinflammatory response accompanies neuronal cell death with microglial cells being the first cell type to be activated (Evans et al., 2013). In human postmortem studies, reactive microglia were detected in regions which play an important role in motor function (motor cortex, motor nuclei of the brainstem, corticospinal tract, spinal cord; Kawamata et al., 1992; Moisse and Strong, 2006). Recently, microglia were also visualized in ALS patients using integrated MR/PET and the radioligand [^11^C]-PBR28 that also binds to TSPO (Alshikho et al., 2016).

While a functional analysis of microglia in living human subjects remains difficult, there are various experimental approaches. In ALS mouse models, microgliosis occurs as well in pre-symptomatic as in symptomatic SOD1.G93A mice (Gerber et al., 2012; Tönges et al., 2014) and in SOD1.G37R mice (Boillée et al., 2006). Here, *in vivo* imaging could demonstrate that microglial cells were highly reactive in pre-symptomatic stages but lost this ability with disease progression (Dibaj et al., 2011). Phenotyping of mSOD1.G93A microglia suggests a predominant M2-type in early onset SOD1.G93A mice and a rather classically activated M1-type at end stage (Liao et al., 2012) indicating that the function of microglia changes during disease progression and both protective or harmful effects may be exerted depending on the particular situation (summarized in Figure 1).

When both, wildtype and mSOD1(G93A) primary microglia were stimulated with LPS, we observed a strong release of pro-inflammatory cytokines and chemokines in both microglial cells types, which was even stronger in the wildtype form (Tönges et al., 2014). This inflammatory response could be effectively attenuated when cells were co-treated with ROCK inhibitor Fasudil. This underlines that ROCK is considerably involved in the modulation of microglial cell function. In line with this, SOD1.G93A mice were shown to exhibit enhanced ROCK activity leading to increased levels of phosphorylated adducin, elevated PTEN activation and decreased Akt activity (Takata et al., 2013). In patients with sporadic ALS, increases of ROCK2 protein have also been shown and were most prominent in skeletal muscle tissue (Conti et al., 2014).

## Rock Inhibition as a Treatment Option for PD and ALS

Therapeutic studies targeting ROCK have been performed in different models of NDD using different strategies to attenuate ROCK activity (reviewed in Hensel et al., 2015). Inhibition of ROCK can be achieved pharmacologically using small molecules targeting either both ROCK isoforms (e.g., Fasudil and Y-27632) or specifically ROCK2 (e.g., SR3677 and SLx-2119; Defert and Boland, 2017). Another approach is the employment of genetic tools that target ROCK expression (e.g., sh- or siRNA).

In PD models, several studies using different approaches of ROCK inhibition have been published (reviewed in Labandeira-Garcia et al., 2015). In a medium severe subchronic MPTP mouse model of PD (30 mg/kg body weight for five consecutive days) oral treatment with Fasudil led to protection of nigral dopaminergic neurons and preservation of the nigrostriatal projections, resulting in an improved motor performance (Tönges et al., 2012). Similar findings were reported in MPTP treated mice (30 mg/kg body weight for five consecutive days) that received the ROCK inhibitor Y-27632 (Villar-Cheda et al., 2012). Interestingly, the effects were attributed both, to a direct neuroprotective effect, which was also present in a microglia-free neuronal culture (Tönges et al., 2012), and to an inhibition of the microglial response (Villar-Cheda et al., 2012). Another study could show that inhibition of the microglial response is essential for the neuroprotective effects of ROCK inhibition in the MPP^+^-mediated dopaminergic degeneration (Borrajo et al., 2014b). Additionally, it was shown that MPTP treatment leads to microglial activation and increased formation of microglia-neuron contacts, so called “gliapses”. Treatment with Fasudil prevented microglial activation and led to increased dopaminergic neuron survival (Barcia et al., 2012).

In another toxin-induced PD model that shows a more severe lesion, the high dose (4 μg/2 μl) striatal 6-OHDA model, oral treatment with Fasudil could not reduce dopaminergic degeneration in mice (Tatenhorst et al., 2014). Using shRNAs that diminished ROCK2 in nigral neurons in the same model, we could show decreased dopaminergic cell death in the substantia nigra. However, striatal fiber density and dopamine contents were not significantly preserved in these animals (Saal et al., 2015). So far, there are no descriptions of the effect of ROCK inhibition on microglial activation in the 6-OHDA model, even though it is known that microglial activation and ROS production contribute to 6-OHDA induced dopaminergic neurodegeneration (Rodriguez-Pallares et al., 2007). The extent of dopaminergic neuron loss in the two described toxin-induced PD models is strongly dependent on the dosage and, in the 6-OHDA model, site of administration. The described studies show that in models with a severe lesion, like the here-described 6-OHDA model, ROCK inhibition alone may not be sufficient to prevent dopaminergic degeneration.

In a transgenic mouse model expressing human mutated α-synuclein (aSyn.A53T), oral Fasudil treatment led to decreased midbrain α-synuclein pathology and improved motor and cognitive functions (Tatenhorst et al., 2016). Even though the microglial contribution to the phenotype was not analyzed here, one could speculate that it plays a role, as aggregated α-synuclein induces microglial “M1” activity (Reynolds et al., 2008; Lee et al., 2010).

Taken together, ROCK inhibition leads to protection of dopaminergic neurons in different models of PD by changing the microglial activation state and exertion of a direct neuroprotective effect.

In ALS, animal studies with ROCK inhibitors are limited to the transgenic SOD1.G93A mouse model. Oral treatment with Fasudil resulted in a slowed disease progression, increased survival time and attenuated motor neuron loss (Takata et al., 2013), which was mediated by decreased PTEN activation and an increase in PI3K-Akt signaling. Similar effects were also observed in our own analysis. Additionally, pre-symptomatically applied Fasudil modified glial responses in SOD1.G93A mice. While astroglial infiltration in the anterior horn was decreased, microglia numbers increased with Fasudil treatment. Furthermore, an *in vitro* activation analysis of primary microglia showed that Fasudil changes the release of cytokines towards an anti-inflammatory profile. Thus, it can be postulated that Fasudil shifts the microglial phenotype towards “M2” in SOD1 mice (Tönges et al., 2014). In animals, where the treatment started with disease onset and thus pathology was already advanced, survival, basic neurological scoring, motoneuron pathology and microglial infiltration could not be significantly improved by Fasudil. However, even in this paradigm the treated animals showed a better motor performance compared to non-treated animals (Günther et al., 2017). In another study, treatment with the 4-aminopyridine ROCK-inhibitor Y-27632 improved motor function in male SOD1.G93A mice (Günther et al., 2014). Since both Fasudil and Y-27632 are not completely selective for ROCK, inhibition of other kinases may also contribute to their effects. Nevertheless, the biological efficacy of these two molecules with chemically distinct back bones strongly argues for ROCK as their main pharmacologically relevant target.

## Pharmacological Rock Inhibitors

In recent years a large number of pharmacological ROCK inhibitors have been developed, most of them belonging either to the chemical group of isoquinoline derivates (e.g., Fasudil; Ripasudil) or aminopyridines (e.g., Y-27632; Feng et al., 2016; Defert and Boland, 2017). The majority of ROCK inhibitors are Type 1 ATP competitive kinase inhibitors that block the transfer of the terminal phosphate from ATP to the respective substrate (Liu and Gray, 2006). In biomedical research Fasudil and Y-27632 are the most extensively used ROCK inhibitors, even though they are less selective against different other kinases and show a limited potency (Feng et al., 2016). Thus, off-target effects that might have contributed to the results have to be considered in studies that used these drugs. Consequently, to date there is only limited data demonstrating the effect of selective or isoform-selective pharmacological ROCK inhibition (reviewed in Defert and Boland, 2017). Development of selective ROCK2 inhibitors would be especially important for the treatment of NDD as ROCK2 is the dominating isoform in the CNS. Furthermore, selective ROCK2 inhibition would minimize the risk of hypotension, a major side effect of long-term treatment with non-selective ROCK inhibitors. So far, two ROCK inhibitors have been licensed for clinical use, both in Japan; Fasudil for the treatment of cerebral vasospasms (Mueller et al., 2005) and Ripasudil for glaucoma treatment (Garnock-Jones, 2014). Possible adverse events under systemic Fasudil treatment are abnormal elevation of liver enzymes (<2%), renal dysfunction (<2%) and hypotension (<5%) that occurred in a small number of treated patients, thus indicating excellent tolerability (Suzuki et al., 2007). In addition, several new compounds are tested in preclinical and clinical trials, exploring the potential of pharmacological ROCK inhibition a promising therapeutic strategy for the treatment of NDD.

## Concluding Remarks and Prospects for Clinical Application

Taken together, there is an obvious microglial contribution to the pathogenesis of PD and ALS and ROCK plays an important role in mediating the microglial inflammatory response. Different studies showed that inhibition of ROCK changes the microglial phenotype from the pro-inflammatory “M1” towards the beneficial “M2” and that it enhances clinically relevant outcomes in models of different NDDs making it an auspicious treatment strategy. Fasudil is already licensed in Japan for the treatment of vasospasms following subarachnoidal hemorrhage and its safety for short-term treatment was proven in several preclinical and clinical trials (Fukumoto et al., 2013; Takata et al., 2013). As NDDs are chronic diseases, long-term effects of systemic ROCK inhibition will have to be evaluated, even though first preclinical long-term studies show excellent tolerability and safety (Tatenhorst et al., 2016). Translational trials in human patients are now needed to evaluate the tolerability, safety and efficacy of ROCK inhibition as a treatment strategy for NDDs.

## Author Contributions

A-ER developed the idea under the lead of PL, performed literature research, wrote and finalized the manuscript and prepared the figure. LT was involved in literature research, writing and revision of the manuscript. PL developed the idea for this review and revised the manuscript. All authors have seen and approved the final version.

## Conflict of Interest Statement

The authors declare that the research was conducted in the absence of any commercial or financial relationships that could be construed as a potential conflict of interest.
